# Relative Motion Estimation Algorithm for Noncooperative Targets Considering Multiple Solutions of Rotational Parameters

**DOI:** 10.3390/s24061811

**Published:** 2024-03-12

**Authors:** Qiyang Hu, Shunan Wu, Fanchen Meng, Zhigang Wu

**Affiliations:** 1 School of Aeronautics and Astronautics, Shenzhen Campus of Sun Yat-sen University, Shenzhen 518107, China; huqy39@mail.sysu.edu.cn (Q.H.); wuzhigang@mail.sysu.edu.cn (Z.W.); 2 Beijing Institute of Aerospace Control Devices, Beijing 100094, China; fanchen_meng@163.com

**Keywords:** relative motion estimation, noncooperative target, on-orbit servicing, Kalman filter

## Abstract

On-orbit servicing using a space robot is gaining popularity among the space community for both economic and safety aspects. In particular, the estimation of the relative motion of a noncooperative target is a challenging problem. This study presents a relative motion estimation scheme based on stereovision for noncooperative targets considering multiple solutions of rotational parameters. Specifically, the mass distribution of the target is identified based on the least-square method and the principle of conservation of angular momentum. Then, the determination of a unique principal axis coordinate frame of the target is employed to resolve the multiple-solution problem. In addition, an EKF (extended Kalman filter)-based filter with global observability is designed to estimate the full motion states and inertia parameters of the target. The convergence performance of the proposed method is verified by numerical simulation. The results also demonstrate that the method is robust to occlusion.

## 1. Introduction

The ever-growing number of malfunctioned spacecrafts remain in orbit with intense space activity, which seriously threatens the safety of operational spacecraft. On-orbit servicing (OOS) technology for repairing, refueling, and deorbiting these defunct spacecrafts has attracted widespread interest in the last decade. Relative pose and motion estimation of the target to be serviced is a key technology in the OOS mission. Frequently, these missions are considered cooperative. In this case, the state of the target can be measured through a global positioning system (GPS) and position-sensing diode (PSD) mounted on the target [1]. However, some defunct spacecrafts are noncooperative targets; i.e., they are unable to actively (i.e., though a communication link) or passively (i.e., though an auxiliary maker) exchange information with the servicing spacecraft, which makes the cooperative architecture inapplicable [2]. Thus, relative motion estimation technology for noncooperative targets has become urgently demanded and challenging.

To address this issue, the servicing spacecraft has to detect the target remotely on its own. Recent work suggests that electronic optical (EO) sensors are the best option for relative motion estimation purposes [3,4] when proximity operation with noncooperative targets is required. Active LIDAR (Light Detection and Ranging) systems and passive monocular/stereovision are typical EO sensors for space application. An LIDAR system can acquire the 3D point cloud of the target, which can be used for motion estimation. The iterative closest point (ICP) may be the most popular algorithm to deal with point clouds for tracking the pose of a target [4]. In [5], the pose is initialized by matching the silhouette image template data with the LIDAR points. The templates are built offline and the sample are restricted to the 2D attitude domain to simplify template matching. Aghili et al. [6] used the pose calculated through the ICP algorithm as the measurement for an extended Kalman filter and derived the covariance of measurement noise. However, LIDAR systems have obvious drawbacks in terms of mass, power consumption, computation load, and hardware complexity, especially for servicing spacecraft with limited weight and energy budget [7]. 

Comparatively, passive sensor-based approaches have been given more attention for noncooperative close-proximity operations. These methods rely on the features of the target surface, which are extracted from a sequence of images, to realize motion estimation. In the single-camera vision system, a model-based estimation architecture is proposed in [8], using the line segments identified from the edge of the target in images. The pose is then computed by solving a feature-matching problem using the efficient perspective-n-point (PNP) method. Since satellite nozzles and docking rings are equivalent to spatial circles, several researchers select circle or ellipse features as the recognized object to estimate the pose of the target [9,10]. However, the symmetry of these features will result in ambiguity of the vector normal and the loss of one rotational degree of freedom [11]. Zhang et al. [12] exploited the elliptical cone model to determine the pose of the docking ring and addressed the duality by introducing images of a redundant nozzle. In [13], a convolutional neural network (CNN) was applied to monocular images for pose determination. Then, an unscented Kalman filter with adaptive process noise was designed to estimate the motion of the target. Nevertheless, reliable datasets with labeled images of different motion states and illumination conditions are required for CNN training, which is costly for space applications. Because monocular vision offers bearing information only, it will suffer scale ambiguity regarding the position magnitude, which limits its application [14]. 

A stereovision system can acquire two perspective views of the features, and therefore, the depth information can be recovered. Several studies have utilized stereovision to address motion estimation concerning uncooperative targets. In [15], the rectangle feature of the framework on the backboard was recognized by two collaborative cameras to realize pose measurement. Hu et al. [16] introduced extra line features to recover information on the roll angle around the circle normal. However, the above methods will not work if particular artificial features, e.g., rectangles, lines, or circles, are not attainable on the target. The point feature always exists on the noncooperative target, making it an ideal candidate feature for recognition, especially when no a priori knowledge of the target’s structure or appearance is accessible [14]. An example of the point-based scheme in which the pose as well as the linear and angular velocity are estimated is shown in [1]. Segal et al. [17] built the observation model of a set of feature points based on the coupling translational–rotational kinematic. Several iterated EKFs with different inertial tensors are exploited and the optimal one is determined by adopting a maximum a posteriori identification. Another work [18] reorganized the Euler equation and incorporated the pseudo-measurement equation into the observation model. 

Since there is no direct information about the target’s attitude in feature point measurement data, it is a crucial aspect to define a target-fixed coordinate frame to describe the orientation of the target. According to recent research work, the principal axis coordinate frame is typically preferred for motion estimation problems of a noncooperative target [6,18,19,20]. However, the principal axis coordinate frame is not unique for a rigid body. If a principal axis coordinate frame is designed as a state to be estimated in the filter without any constraint, it will bring multiple solution problems to angular velocity, the inertia matrix, and coordinates of features, because these values completely depend on which coordinate frame is utilized to describe the target’s rotation. In other words, multiple sets of these rotational parameters will share the same measurement history, resulting in the lack of global observability of the estimation problem [21]. The multiple-solution problem of rotational parameters, to the knowledge of the authors, has not been mentioned and investigated in the literature concerning relative navigation. Another aspect to take into account is that the rotation of the target will inevitably lead to the occlusion of feature points. During the occlusion period, the estimates will solely rely on the propagation of the dynamic model until these feature points become visible to the sensor again. In this circumstance, the filter may suffer serious convergence problems if the global observability cannot be guaranteed, making it vulnerable to occlusion. 

Motivated by this, this study developed a relative motion estimation algorithm for noncooperative targets considering multiple solutions of rotational parameters. The method proposed herein only depends on the tracking of feature points by using stereovision measurements and prior information about the geometric shape of the target is not required. The original contributions of our work are twofold: First, we propose a method to determine the attitude of the target, which has a unique solution of the principal axis coordinate frame. Second, we use EKF along with a uniquely determined principal axis coordinate frame to guarantee global observability. In numerical simulation, the robustness of the algorithm to occlusion is presented and validated.

The rest of the article is organized as follows: Section 2 introduces the observation model of the stereovision system, as well as the dynamic model of the noncooperative target. Section 3 illustrates the multiple-solution problem of rotational parameters in detail and introduces the method for the determination of the principal axis coordinate frame. Section 4 formulates the EKF-based filtering scheme with a determined principal axis coordinate frame. Then, in Section 5, the simulation results are presented. Finally, a conclusion is drawn in Section 6.

## 2. Mathematical Model

The aim of the relative motion estimation problem is to estimate the relative translational and rotational motion states and inertial parameters of noncooperative targets using the stereovision equipped on the servicing satellite. In this section, the system model, namely, the measurement model of the stereovision and the dynamic model of the target, are presented. Several coordinate systems are introduced to help describe these models.

### 2.1. Measurement Model of Stereovision

**Figure 1:**
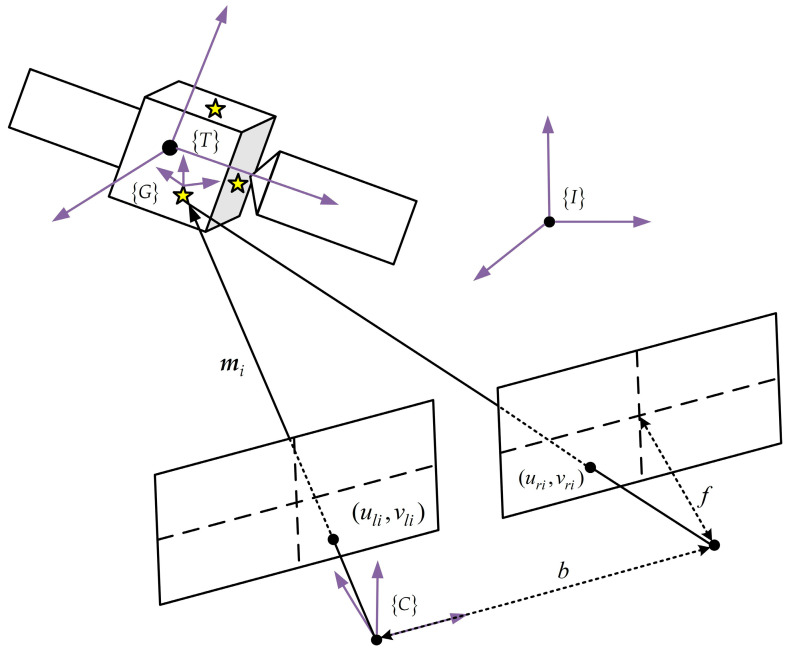
Diagram of stereovision system.

As depicted in Figure 1, a stereovision system is employed on the space robot to observe the target. A simplified measurement model is applied, characterized by two parallel image planes that are perpendicular to the optical axis. Let C denote the sensor coordinate frame which is attached to the center of projection of the left camera. The *x* axis of frame C point to the center of projection of the right camera, the *y* axis is aligned with the optical axis, and the *z* axis obeys the right-hand rule. It is assumed that N feature points on the surface of the target can be detected. The projection of the i th feature points in the left and right image planes is denoted as $\left(u_{il},v_{il}\right)$ and $\left(u_{ir},v_{ir}\right)$, respectively. Then, the coordinate expressed in frame C can be recovered using a pinhole camera model [17]:

(1) $$m_{i}=\left[\begin{array}{ccc} x_{i} & y_{i} & z_{i} \end{array}\right]=\left[\begin{array}{ccc} z_{i}\frac{u_{il}}{f} & z_{i}\frac{v_{il}}{f} & \frac{fb}{u_{il}-u_{ir}} \end{array}\right]$$

where f is the focal length and b is the baseline length.

### 2.2. Dynamic Model of Rotational Motion

In this article, the attitude of the target is parameterized using a quaternion. The kinematics of the quaternion is given as [22,23]

(2) qIG=12ωGIG0⊗qIG

where qIG describes the orientation of any target-fixed coordinate frame (G) with respect to the inertial coordinate frame, I. ωGIG is the angular velocity of frame G with respect to frame I expressed in G, and ⊗ represents the quaternion multiplication. When the target is regarded as a rigid body, the rotational dynamics is given as [6]

(3) $$I\dot{\omega }=-\left[\omega \times \right]I\omega +L$$

where I denotes the target’s inertia matrix and L is external disturbance torque.

### 2.3. Dynamics Model of Translational Motion

Assuming that the servicing spacecraft moves in a circular orbit, the translational motion of the target in the Hill coordinate frame of the space robot (denoted by L) can be described by the Hill equation [24]:

(4) $$\left[\begin{array}{c} \dot{r}\\ \dot{v} \end{array}\right]=\left[\begin{array}{cc} 0_{3\times 3} & I_{3\times 3}\\ E_{1} & E_{2} \end{array}\right]\left[\begin{array}{l} r\\ v \end{array}\right]+\left[\begin{array}{l} 0_{3\times 3}\\ I_{3\times 3} \end{array}\right]n_{t}$$

where

(5) $$E_{1}=\left[\begin{array}{ccc} 0 & 0 & 0\\ 0 & -{\omega_{c}}^{2} & 0\\ 0 & 0 & 3{\omega_{c}}^{2} \end{array}\right]E_{2}=\left[\begin{array}{ccc} 0 & 0 & 2\omega_{c}\\ 0 & 0 & 0\\ -2\omega_{c} & 0 & 0 \end{array}\right]$$

$r={\left[\begin{array}{ccc} x & y & z \end{array}\right]}^{T}$ and $v={\left[\begin{array}{ccc} \dot{x} & \dot{y} & \dot{z} \end{array}\right]}^{T}$ are the relative position and velocity of the target with respect to the servicing satellite expressed in frame L. $\omega_{c}$ is the orbital angular velocity of the servicing spacecraft.

## 3. Determination of Principal Axis Coordinate Frame 

**Figure 2:**
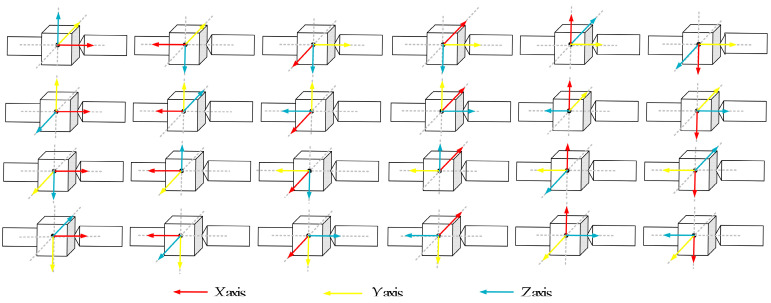
Multiple solutions of principal axis coordinate frame.

As mentioned in the introduction, the principal axis coordinate frame, the target-fixed coordinate frame aligned with its principal axis of inertia, is more preferable to describe the orientation of a noncooperative target and often set as the state to be estimated in a filter. One reason is that the principal axis coordinate frame can reflect the mass distribution of the target, which is useful information for the subsequent design of the capture strategy. Moreover, because the corresponding inertia matrix is diagonal, it will reduce the dimension of unknown inertia parameters. Notice that there are different ways to define a principal axis coordinate frame. (Figure 2 shows a total of 24 principal axis coordinate frames for a rigid body. The principal axes of the target are represented by dashed lines.) Because the principal axis is only determined by the mass property of the target, it is usually unable to be directly measured by the optical sensor. In other words, the target-fixed frame, G, which is related to visual measurement, does not coincide with a principal axis frame in general. As shown in Figure 2, the attitudes of these principal axis coordinate frames are different from each other, which further causes different values of the corresponding angular velocity, inertial matrix, and coordinates of features. These 24 sets of rotational parameters can produce the same time history of measurement and lead to a multiple-solution problem. Consequently, the navigation filter will lose global observability. 

Motivated by this, an algorithm for the determination of a unique principal axis coordinate frame is proposed in this section. In this phase, three non-collinear feature points are exploited to define frame G due to the lack of direct pose measurement of the target. The coordinates of these points are computed based on Equation (1). Three orthogonal unit vectors, $c_{i}$, can be obtained using the following equation:

(6) $$\left\{\begin{array}{l} c_{1}=\left(m_{2}-m_{1}\right)/\left|\left(m_{2}-m_{1}\right)\right|\\ c_{3}=\left(m_{2}-m_{1}\right)\times \left(m_{3}-m_{1}\right)/\left|\left(m_{2}-m_{1}\right)\times \left(m_{3}-m_{1}\right)\right|\\ c_{2}=c_{3}\times c_{1} \end{array}\right.$$

Then, a target-fixed photogrammetric coordinate frame, G, is defined, which conforms to

(7) RCG=c1c2c3T

where RCG is the rotation matrix from frame C to frame G. The corresponding inertial matrix expressed in frame G is denoted as

(8) IG=IxxIxyIxzIxyIyyIyzIxzIyzIzz

Since these feature points are randomly distributed and selected, the product of inertia is set as non-diagonal elements of the inertia matrix without loss of generality. It is worth noting that only five inertial parameters are independent because the inertial matrix will always conform to Equation (3), even when multiplied by any constant. After being divided by the first element, the inertial matrix can be normalized as

(9) I¯G=1I¯xyI¯xzI¯xyI¯yyI¯yzI¯xzI¯yzI¯zz

and the constant inertia ratio vector is expressed as $l={\left[\begin{array}{ccccc} {\bar{I}}_{yy} & {\bar{I}}_{zz} & {\bar{I}}_{zy} & {\bar{I}}_{xz} & {\bar{I}}_{yz} \end{array}\right]}^{T}$. According to attitude dynamics, the angular momentum of target can be formulated as

(10) I¯Gω=RIGH

where $H={\left[\begin{array}{ccc} h_{1} & h_{2} & h_{3} \end{array}\right]}^{T}$ denotes the angular momentum expressed in frame I. It is assumed that the target is a torque-free tumbling rigid body; the principle of the conservation of angular momentum can be adopted to estimate the inertia parameters. In that case, H will remain constant. Consequently, Equation (10) can be rewritten as the following linear equation:

(11) a(t)X=b(t)

where

(12) a(t)=00ωy(t)ωz(t)0ωy(t)0ωx(t)0ωz(t)−RIG(t)0ωz(t)0ωx(t)ωy(t),b(t)=−ωx00

$X={\left[\begin{array}{cc} l^{T} & H^{T} \end{array}\right]}^{T}$. The unknown constant X is estimated by the least-square method if observation data from different epochs are acquired:

(13) $$\hat{x}=(A^{T}A{)}^{-1}A^{T}B$$

where

(14) $$A=\left[\begin{array}{c} a(t_{1})\\ a(t_{2})\\ \vdots \\ a(t_{k}) \end{array}\right],B=\left[\begin{array}{c} b(t_{1})\\ b(t_{2})\\ \vdots \\ b(t_{k}) \end{array}\right]$$

To estimate the angular velocity in Equation (12), we rewrite Equation (2) as

(15) $$\omega =2\Xi^{T}\left(q\right)\overset{.}{q}$$

where

(16) $$\Xi (q)\equiv \left[\begin{array}{c} q_{4}I_{3}+\left[q_{1:3}\times \right]\\ -q_{1:3}^{T} \end{array}\right]$$

The angular velocity can be approximated from the numerical differentiation of q [25]. 

The resulting $\hat{\bar{I}}$ relates the mass distribution of the target to the stereovision measurement. Therefore, the orientation of a principal axis coordinate frame, T, can be determined through orthogonal diagonalization of $\bar{I}$:

(17) RGTI¯^GRGTT=r1r2r3TI¯^Gr1r2r3=λ1λ2λ3

where $\lambda_{i}$ are eigenvalues of $\bar{I}$. The corresponding eigenvectors, $r_{i}$, are the column vectors of the rotation matrix from frame T to frame *G*. Note that multiple solutions of frame T exist, resulting from the selection of $r_{i}$, as shown in Figure 2. Therefore, an approach to uniquely determine frame *T* is proposed as follows: Specifically, $r_{i}$ is chosen so that the inequality constraint $\lambda_{1}>\lambda_{2}>\lambda_{3}$ can be satisfied. Furthermore, the first element of $r_{1}$ is set to be positive. In fact, these conditions are equivalent to the constraints that the *x* axis and *z* axis of frame T are aligned with the principal axis of the largest and smallest moment of inertia, respectively, and that the *x* axis of frame T forms an acute angle with the *x* axis of frame G. In this way, the principal axis coordinate frame, *T*, can be uniquely determined. 

## 4. Extended Kalman Filter with Determined Principal Axis Coordinate Frame 

In our implementation, an EKF-based scheme is employed using the estimated attitude of frame T and observed feature points to estimate the rotational and translational motion of the target. Meanwhile, the orientation of frame T is set as the rotational state of the target to be filtered. Because frame T has been defined and a rough estimation is directly acquired in Section 3, the multiple-solution problem will be resolved. 

We denote the inertia matrix of the principal axis coordinate frame, T, as

(18) $$I=\left[\begin{array}{ccc} I_{x} & 0 & 0\\ 0 & I_{y} & 0\\ 0 & 0 & I_{z} \end{array}\right]$$

which is parameterized in a similar way as in [6].

(19) $$p_{x}=\frac{I_{y}-I_{z}}{I_{x}},p_{y}=\frac{I_{z}-I_{x}}{I_{y}},p_{z}=\frac{I_{x}-I_{y}}{I_{z}}$$

where $p={\left[\begin{array}{ccc} p_{x} & p_{y} & p_{z} \end{array}\right]}^{T}$ is the inertia ratio with

(20) $$\dot{p}=0$$

Therefore, Euler dynamics (3) can be rewritten in terms of the inertia ratio as

(21) $$\dot{\omega }=K(\omega )+J(p)n_{r}$$

where

(22) $$K(\omega )=\left[\begin{array}{l} p_{x}\omega_{y}\omega_{z}\\ p_{y}\omega_{x}\omega_{z}\\ p_{z}\omega_{x}\omega_{y} \end{array}\right],J(p)=\left[\begin{array}{ccc} 1 & 0 & 0\\ 0 & \frac{1-p_{y}}{1+p_{x}} & 0\\ 0 & 0 & \frac{1+p_{z}}{1-p_{x}} \end{array}\right]$$

$n_{r}$ is the disturbance torque. 

Let $f_{i}$ denote the coordinate of feature points in frame T, which is constant, i.e.,

(23) $${\dot{f}}_{i}=0$$

The state vector to be estimated by the filter is therefore

(24) x=[qITTωTpTrTvTf1Tf2T⋯fNT]T

From Equations (2), (4), (20), (21) and (23), the system model is described by

(25) x=f(x)

Considering the composition rules of the quaternion, the error-state vector is given by

(26) $$\Delta x={\left[\begin{array}{ccccccccc} \delta \theta^{T} & \delta \omega^{T} & \delta p^{T} & \delta r^{T} & \delta v^{T} & \delta f_{1}^{T} & \delta f_{2}^{T} & \cdots & \delta f_{N}^{T} \end{array}\right]}^{T}$$

For the angular velocity, inertia ratio, position, velocity, and coordinates of feature points, the error in the estimated $\hat{b}$ of a state (b) is defined as $\delta b=b-\hat{b}$. For the quaternion, the error is parameterized using a rotation vector (δθ) which satisfies

(27) $$q=\left[\begin{array}{c} \frac{1}{2}\delta \theta \\ \sqrt{1-{\left\|\frac{1}{2}\delta \theta \right\|}^{2}} \end{array}\right]⊗\hat{q}$$

The linearized continuous-time model for the error states is obtained by retaining only the first-order term of the Taylor expansion of Equation (25) around the current estimated value [22]:

(28) $$\Delta \dot{x}=F\Delta x+Gn$$

where

(29) $$F=\left[\begin{array}{cccccc} -[\hat{\omega }\times ] & I_{3} & 0_{3\times 3} & 0_{3\times 3} & 0_{3\times 3} & 0_{3\times 3N}\\ 0_{3\times 3} & M(\hat{\omega },\hat{p}) & N(\hat{\omega }) & 0_{3\times 3} & 0_{3\times 3} & 0_{3\times 3N}\\ 0_{3\times 3} & 0_{3\times 3} & 0_{3\times 3} & 0_{3\times 3} & 0_{3\times 3} & 0_{3\times 3N}\\ 0_{3\times 3} & 0_{3\times 3} & 0_{3\times 3} & 0_{3\times 3} & I_{3\times 3} & 0_{3\times 3N}\\ 0_{3\times 3} & 0_{3\times 3} & 0_{3\times 3} & E_{1} & E_{2} & 0_{3\times 3N}\\ 0_{3N\times 3} & 0_{3N\times 3} & 0_{3N\times 3} & 0_{3N\times 3} & 0_{3N\times 3} & 0_{3N\times 3N} \end{array}\right],G=\left[\begin{array}{cc} 0_{3\times 3} & 0_{3\times 3}\\ J(\bar{p}) & 0_{3\times 3}\\ 0_{3\times 3} & 0_{3\times 3}\\ 0_{3\times 3} & 0_{3\times 3}\\ 0_{3\times 3} & I_{3}\\ 0_{3N\times 3} & 0_{3N\times 3} \end{array}\right]$$

$n={\left[\begin{array}{cc} n_{r}^{T} & n_{t}^{T} \end{array}\right]}^{T}$ with

(30) $$M(\hat{\omega },\hat{p})=\left[\begin{array}{ccc} 0 & p_{x}\omega_{z} & p_{x}\omega_{y}\\ p_{y}\omega_{z} & 0 & p_{y}\omega_{x}\\ p_{z}\omega_{y} & p_{z}\omega_{x} & 0 \end{array}\right],N(\hat{\omega })=\left[\begin{array}{ccc} \omega_{y}\omega_{z} & 0 & 0\\ 0 & \omega_{x}\omega_{z} & 0\\ 0 & 0 & \omega_{x}\omega_{y} \end{array}\right]$$

The covariance of process noise (n) is denoted by Q.

As mentioned above, the estimated attitude of frame T in Section 3 offers the direction observation of the state within the filter, the measurement model of which is simply

(31) y0=qIT=qCT⊗qIC

where qIC is computed based on the attitude installation matrix of stereovision and the attitude estimation of the servicing spacecraft. Meanwhile, the coordinate of the i th feature point in frame L satisfies

(32) yi=r+RILRTIfi

which is obtained from the absolute orbit determination of the servicing spacecraft. Putting all the components together, the observation model of filter is defined as

(33) $$y=h(x)={\left[\begin{array}{ccccc} y_{0}^{T} & y_{1}^{T} & y_{2}^{T} & \cdots & y_{N}^{T} \end{array}\right]}^{T}$$

The error measurement model is approximated by linearizing Equation (33):

(34) Δy=HΔx+η

where η is the measurement noise, and the measurement sensitive matrix (H) is given as

(35) H=I3×303×303×303×303×303×3−R^TL[f^1×]03×303×3I3×303×3R^TL⋯⋯⋯⋯⋯⋯−R^TL[f^N×]03×303×3I3×303×3R^TL

In this study, a continuous–discrete type of EKF is employed to solve the relative motion estimation problem. The implementation of the EKF is based on two processes: prediction and update. 

In the prediction step, the optimal estimation of the state (*x*) and error covariance (*P*) are propagated for the time interval $\left(t_{k},t_{k+1}\right)$ through

(36) $$x_{k+1}^{-}=x_{k}^{+}+\int_{t_{k}}^{t_{k+1}}f(x(t))dt$$

(37) $$P_{k+1}^{-}=\Phi (t_{k+1},t_{k})P_{k}^{+}\Phi^{T}(t_{k+1},t_{k})+Q_{k}$$

The error-state transition matrix $\Phi (t_{k+1},t_{k})$ can be computed by numerical simulation of the following differential equation:

(38) $$\dot{\Phi }(\tau ,t_{k})=F(\tau )\Phi (\tau ,t_{k})$$

With the initial condition of $\Phi (t_{k},t_{k})=I$. $Q_{k}$ is the discrete covariance of system noise and is calculated by [26]

(39) $$Q_{k}=\int_{t_{k}}^{t_{K+1}}\Phi (t_{k+1},\tau )GQG^{T}\Phi^{T}(t_{k+1},\tau )d\tau$$

Once the measurement is available, the error state (Δx) and the corresponding covariance are corrected according to

(40) $$\Delta x_{k+1}=K_{k+1}\Delta y_{k+1}$$

(41) $$P_{k+1}^{+}=\left(I-K_{k+1}H_{k+1}\right)P_{k+1}^{-}{\left(I-K_{k+1}H_{k+1}\right)}^{T}+K_{k+1}R_{k+1}K_{k+1}^{T}$$

where $R_{k+1}$ is the covariance of measurement noise and $K_{k+1}$ is the Kalman gain.

(42) $$K_{k+1}=P_{k+1}^{-}H_{k+1}^{T}{\left(H_{k+1}P_{k+1}^{-}H_{k+1}^{T}+R_{k+1}\right)}^{-1}$$

The optimal estimation of filter states, except the attitude, can be updated as

(43) $$x_{k+1}^{+}=x_{k+1}^{-}+\Delta x_{k+1}$$

To satisfy the unit norm constraint, the update of quaternion is realized through

(44) $$q_{k+1}^{+}=\left[\begin{array}{c} \frac{1}{2}\delta \theta_{k+1}\\ \sqrt{1-{\left\|\frac{1}{2}\delta \theta_{k+1}\right\|}^{2}} \end{array}\right]⊗q_{k+1}^{-}$$

## 5. Numerical Simulation

In this section, the numerical simulations are carried out to verify the proposed relative motion estimation method. Specifically, the objectives of the simulative experiment are to (a) evaluate the validity of the determination of the principal axis coordinate frame and (b) investigate the estimation performance of the EKF-based filter with a uniquely determined attitude. 

**Figure 3:**
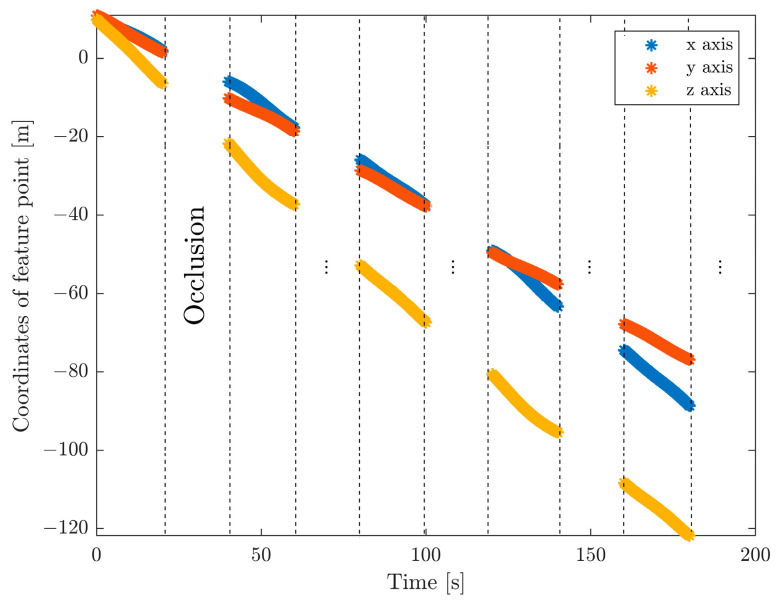
Raw coordinate information of feature points in the presence of occlusion.

**Figure 4:**
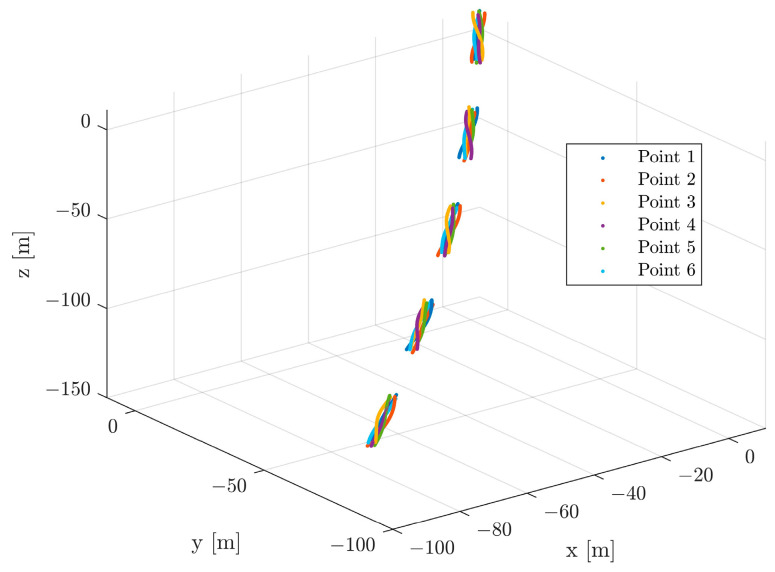
Visible trajectories of six different feature points.

It is assumed that the servicing spacecraft is in a circular orbit with a radius of 6800 km, and thus, the angular rate is 0.0012 rad/s. The initial relative position and velocity of the target with respect to the servicing spacecraft are set as $r_{0}={\left[10,10,10\right]}^{T}$ m and $v_{0}={\left[-0.3889,-0.4932,-0.8264\right]}^{T}$ m/s. In this simulation, a microsatellite, mentioned in [6], is selected as the target spacecraft, which has the inertia matrix $\mathrm{diag}([8,5,4])\;\mathrm{kg}\cdot m^{2}$. The initial attitude of the target parameterized by the quaternion is given as $q_{0}={\left[0,0,0,1\right]}^{T}$. According to [27], a malfunctional spacecraft may tumble at a rate varying greatly, from 2.9 deg/s to 36 deg/s. Based on this, a typical value of $w_{0}={\left[0.1,0.1,0.1\right]}^{T}$ rad/s is considered as the initial angular velocity. We assume that the coordinates of feature points expressed in frame T are subject to uniform distribution, with lower and upper bounds of −1.5 m and 1.5 m, which is the same as in [17]. Six instead of ten feature points are supposed to be measured to test the proposed method in extreme conditions. The measurement is generated at a rate of 10 Hz (similarly to [28]). Due to the self-occlusion of the target caused by rotation, these feature points will inevitably be unobservable for some time intervals. The availability of a feature point can be identified by evaluating whether the angle between the observation direction and the normal direction is acute [29]. However, this algorithm is reliable only when the body is convex. For simplicity, a hypothesis of a fixed occlusion period is made, which is sufficient for the purpose of this study. In view of the magnitude of the initial angular rate, $\left|w_{0}\right|\approx 10$ deg/s, we consider a complete unavailability of measurement within 20 s, about half of the rotation period of the target. Figure 3 illustrates this concept. In addition, Figure 4 shows the visible trajectories of these six feature points.

(1) Results of principal axis coordinate determination

This section presents the principal axis coordinate determination results. To evaluate the influence of the measurement noise level, we consider the stereovision system with an angular resolution of $0.1\times 10^{-5}\mathrm{rad}$, $0.5\times 10^{-5}\mathrm{rad}$, $2\times 10^{-5}\mathrm{rad}$, and $5\times 10^{-5}\mathrm{rad}$, similar to the setup used in [30]. The root mean square error (RMSE) is applied as the metric of the algorithm, which is defined as

(45) $$\mathrm{RMSE}=\sqrt{\frac{1}{N}\sum_{i=1}^{N}e_{i}^{T}e_{i}}$$

where e is the estimated error and N is the number of Monte Carlo runs.

**Figure 5:**
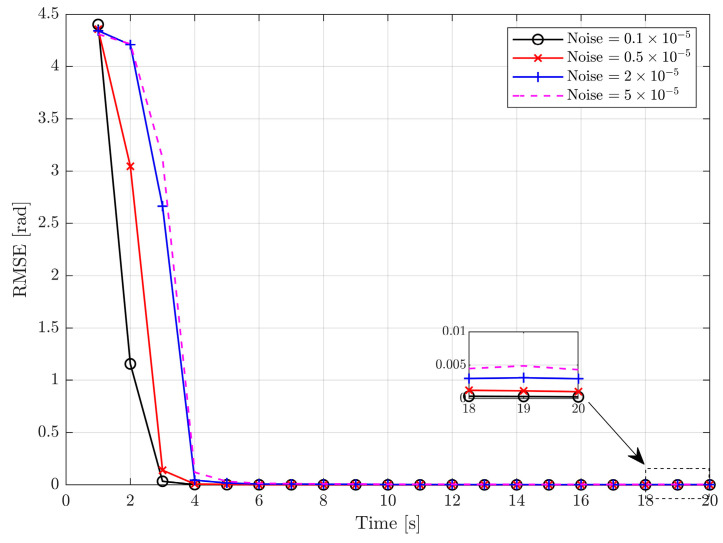
RMSE of attitude determination of principal axis coordinate frame.

The RMSE of the estimated attitude of the principal axis coordinate frame is shown in Figure 5, with different levels of noise over 500 Monte Carlo runs. As seen, all RMSE plots at different noise levels converge quickly in a few seconds with acceptable performance, even before the occurrence of occlusion. It is clearly shown that the noise covariance variations have a great influence on the accuracy and convergence performance: an increased noise level implies lower accuracy and slower convergence. This is because the determination of the principal axis coordinate frame is based on the identification and diagonalization of the inertial matrix, the accuracy of which is directly related to the measurement accuracy. Overall, it is safe to state that the principal axis coordinate frame can be determined uniquely with relatively high accuracy through the proposed algorithm, which is used during the subsequent filter phase.

(2) Results of motion estimation

In this section, the EKF with the determination of the principal axis coordinate frame (DPACF) is implemented to estimate the motion of the target. The covariance of the orbital force and disturbance torque are set as $\sigma_{t}^{2}=2\times 10^{-6}I_{3}\;m^{2}{/s}^{4}$ and $\sigma_{r}^{2}=2.5\times 10^{-5}I_{3}\;{\mathrm{rad}}^{2}{/s}^{4}$, the same as in [6]. Note that the states and parameters are predicted by solely relying on their latest values and propagation of dynamics during the occlusion period shown in Figure 3. We evaluate the performance of the proposed scheme by comparing it with the method in [6], not employing DPACF. The simulation time is 200 s.

**Figure 6:**
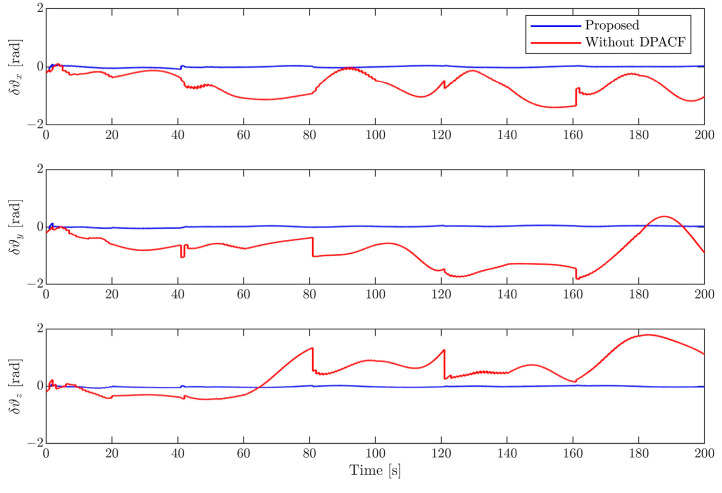
Estimation error of attitude angle.

**Figure 7:**
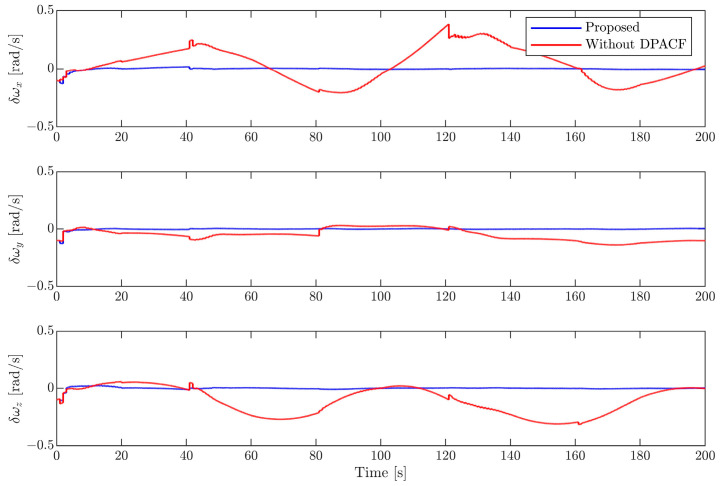
Estimation error of angular velocity.

**Figure 8:**
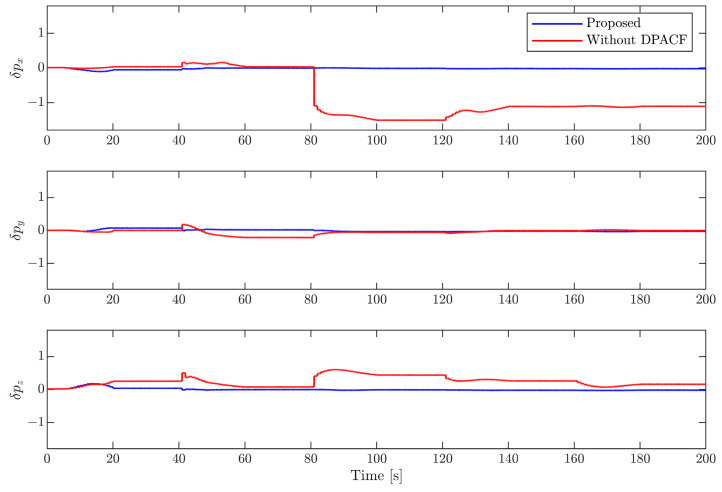
Estimation error of inertia ratio.

**Figure 9:**
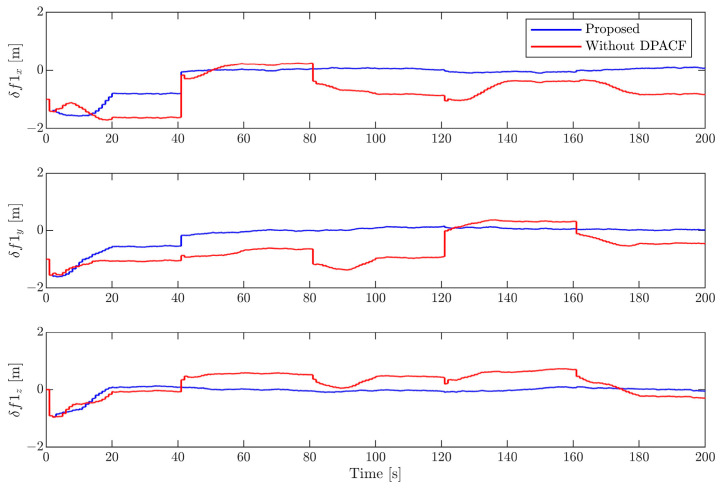
Estimation error of coordinates of feature point.

**Figure 10:**
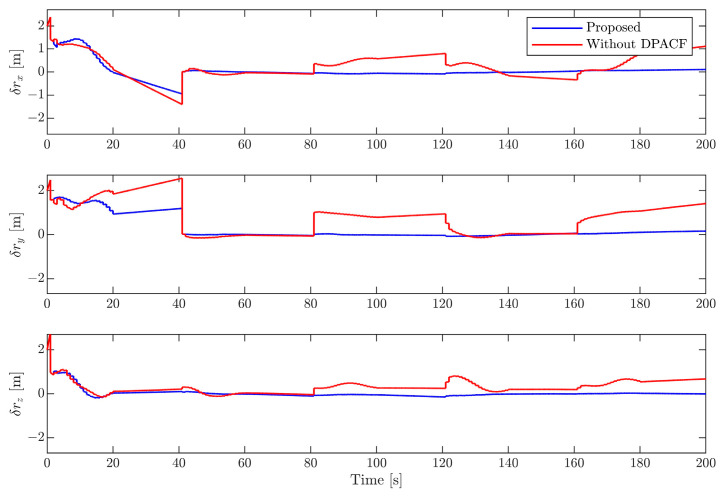
Estimation error of relative position.

**Figure 11:**
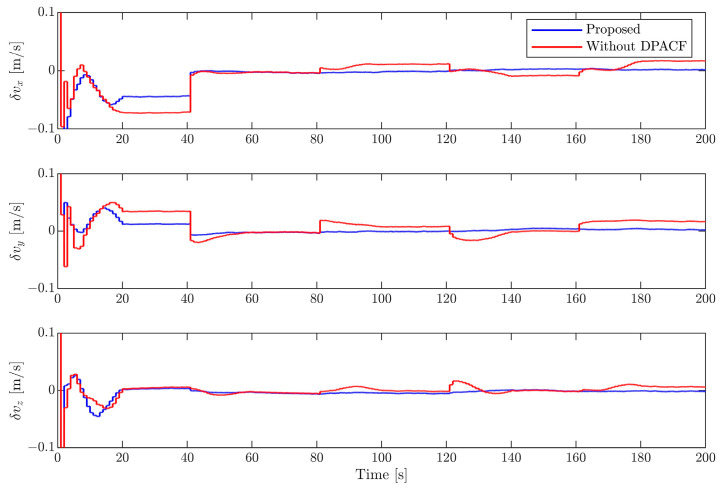
Estimation error of relative velocity.

Figure 6, Figure 7, Figure 8 and Figure 9 show the estimation errors of rotational parameters, i.e., the attitude angle, angular velocity, inertia ratio, and coordinates of feature points. It is evident from the figure that estimation errors of the method without DPACF are characterized by significant nonzero mean deviation in the whole process. This is because, due to the multiple solutions of the principal axis coordinate frame, the rotational parameters are not unique. In other words, the filter is not globally observable. On the other hand, when the feature points are missing during occlusion, the noise process will lead to a deviation of estimates from the solution it is supposed to converge to. Therefore, there is no guarantee that the filter will converge to the previous or any specific solution of the 24 candidates in the next estimation phase when the measurements are available again, which results in the fluctuation of estimates. This makes the estimation process fragile to occlusion. Comparatively, the error curves of our method can converge in some periods of time and finally remain within a small neighborhood around zero. This mainly benefits from the determination of the principal axis coordinate frame to force the filter to converge to a unique solution, defined in Section 3. The estimation errors of the relative position and velocity are shown in Figure 10 and Figure 11. It can be seen that the divergence of rotational parameters even causes translational states to deviate, although there is no multiple-solution problem in these states. In conclusion, the proposed method is expected to remove the multiple-solution problem and exhibit robustness to inevitable occlusion. 

## 6. Conclusions

A relative motion estimation scheme for noncooperative targets considering multiple solutions of rotational parameters is presented in this paper. This scheme depends on the determination of the principal axis coordinate frame before the implementation of a filter. Through the identification of the mass distribution of the target and the diagonalization of the normalized inertia matrix, the multiple-solution problem of rotational parameters is prohibited and the global observability of the estimation problem is guaranteed. The results demonstrate good convergence performance and robustness to occlusion, owing to the new filter structure with a unique solution. It should be noted that such an algorithm improves the estimation capability and provides a feasible solution for the relative motion estimation problem of future OOS missions.

## Data Availability

Data are contained within the article.
